# Case Report: Rowell Syndrome–Like Flare of Cutaneous Lupus Erythematosus Following COVID-19 Infection

**DOI:** 10.3389/fmed.2022.815743

**Published:** 2022-02-14

**Authors:** Kossara Drenovska, Martin Shahid, Valeria Mateeva, Snejina Vassileva

**Affiliations:** Department of Dermatology and Venereology, Medical University-Sofia, Sofia, Bulgaria

**Keywords:** COVID-19, Rowell syndrome, erythema multiforme-like, subacute cutaneous lupus erythematosus, flare

## Abstract

The current COVID-19 pandemic caused by severe acute respiratory syndrome coronavirus 2 (SARS-CoV-2) has had an important impact on dermatology practice, posing diagnostic and therapeutic challenges especially in patients with inflammatory and autoimmune skin disorders. Disease-specific and nonspecific cutaneous manifestations have been increasingly reported in the spectrum of COVID-19 but the influence of the infection on pre-existing dermatologic diseases has not been clearly defined. There has been a debate in the literature as to whether patients suffering from autoimmune dermatoses, including cutaneous lupus erythematosus (CLE), are at increased risk of SARS-CoV-2 infection, as well as if they experience worsening of their lupus erythematosus (LE)-related clinical symptoms. This article reports on a case of Rowell syndrome occurring after COVID-19 in a 67-year old woman with pre-existing chronic CLE manifesting with few discoid lesions on the face, scalp, and upper chest, successfully controlled with topical corticosteroids and photoprotection. Erythema multiforme (EM)-like eruption developed approximately two weeks after the SARS-CoV-2 infection, the latter being confirmed by positive nasopharyngeal swab and successfully treated with systemic antibiotics and antiaggregants. Diffuse hair loss and patches of cicatricial alopecia were also present upon scalp examination. Laboratory workup, including routine tests, histologic, immunofluorescent, and serologic investigations, was supportive to the diagnosis. Administration of topical and systemic corticosteroids along with peroral hydroxychloroquine resulted in the progressive improvement of the cutaneous lesions. Rowell syndrome is a rare entity in the spectrum of LE, characterized by EM-like lesions, photosensitivity, and positive antinuclear and anti-Ro antibodies, that is currently considered to be a variant of subacute CLE (SCLE). Several cases of SCLE have been described in association with medications, including anti-SARS-CoV-2 vaccines but only a few reports incriminate the infection itself as a potential exacerbating factor. Based on the clinical course of the disease, we suggest that the observed Rowell syndrome-like flare of CLE was related to the COVID-19 infection in this patient.

## Introduction

The outbreak of COVID-19 caused by severe acute respiratory syndrome coronavirus 2 (SARS-CoV-2) has had an important impact on dermatology practice, posing diagnostic and therapeutic challenges especially in patients with inflammatory and autoimmune skin disorders. Disease-specific and non-specific cutaneous manifestations have been increasingly reported in the spectrum of COVID-19 but the influence of the infection on pre-existing dermatologic diseases has not been clearly defined. There has been a debate in the literature as to whether patients suffering from autoimmune skin diseases, including the various subtypes in the broad spectrum of cutaneous lupus erythematosus (CLE), are at increased risk of SARS-CoV-2 infection, as well as if they experience worsening of their lupus erythematosus (LE)-related clinical symptoms. In recent months, there have been multiple publications in the form of case reports, case series, observational and retrospective studies on COVID-19 in patients with systemic LE (1); however, not much information is present in the literature on the effect of coronavirus infection on multiple subtypes and clinical variants of CLE including chronic CLE and subacute CLE (SCLE). Though skin manifestations of COVID-19 are rare, they are diverse (2) and some of them might create confusion with the wide range of skin changes in the spectrum of CLE (3). In this respect, it is of interest to record all cases of occurrence of SARS-CoV-2 infection in the setting of cutaneous lupus and vice versa.

We present a case of Rowell syndrome-like flare of CLE following COVID-19.

## Case Report

A 67-year-old Caucasian woman was admitted to our Dermatology department in April 2021 for a non-pruritic and slightly burning erythematous and scaly cutaneous eruption affecting sun-exposed areas that started two weeks after a mild COVID-19. The patient reported that she had been suffering from “photosensitivity” since her young age and had been diagnosed 6 years ago with chronic CLE manifesting with few discoid lesions on the face, scalp, and upper chest, successfully controlled with topical corticosteroids and photoprotection. In February 2021 she experienced intermittent fever up to 38°C, dry cough, and malaise. Reverse-transcription PCR (RT-PCR) on a nasopharyngeal swab tested positive for SARS-CoV-2 but there was no need for hospital care because of the patient's good general condition with oxygen saturation within normal limits and no signs of pneumonia. Therefore, she underwent outpatient quarantine and peroral treatment with azithromycin.

**Figure 1:**
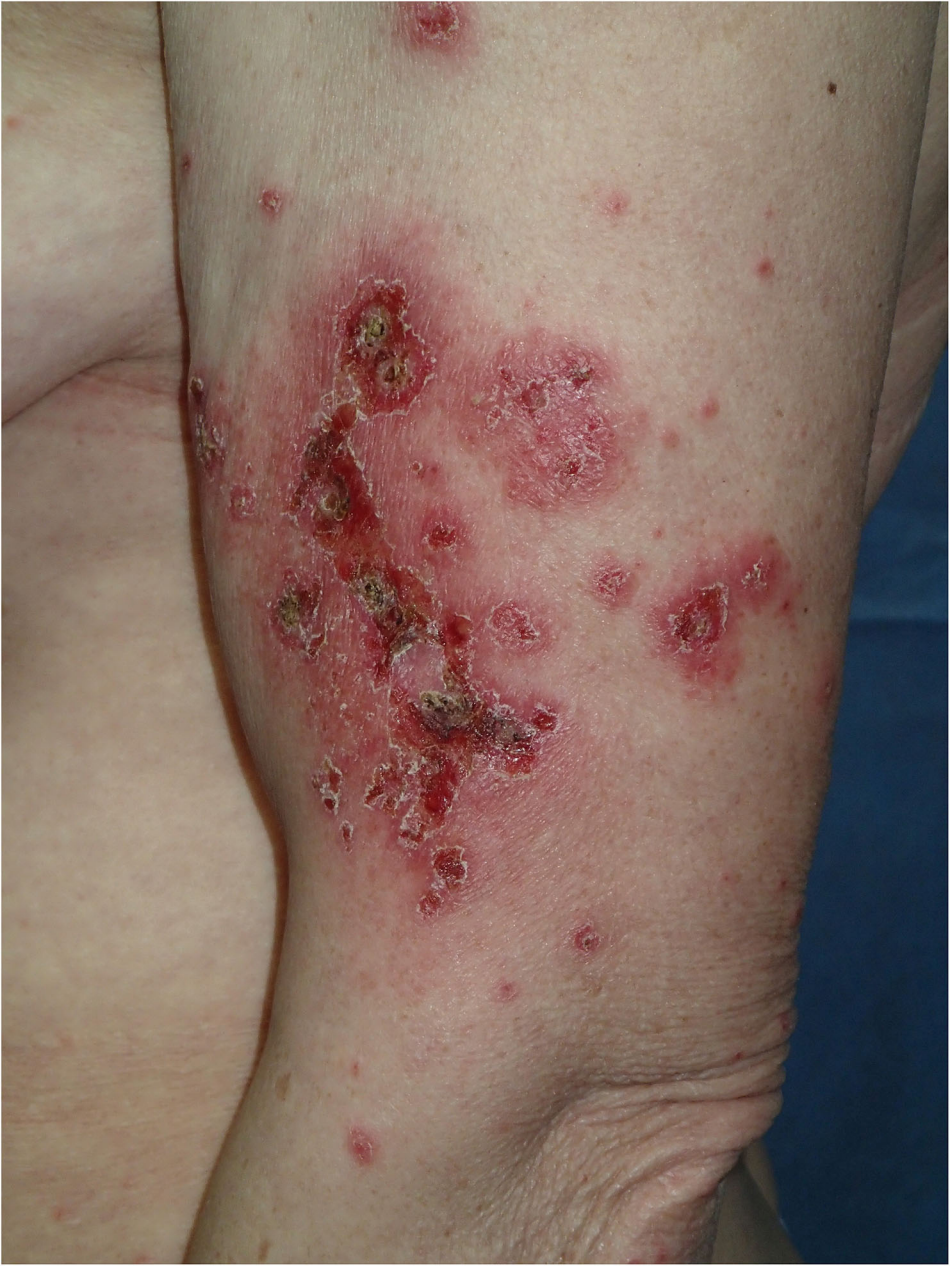
Annular-polycyclic photosensitive eruption of subacute cutaneous lupus erythematosus (SCLE) occurring after COVID-19 illness; note the trailing scale in the periphery of the lesions.

**Figure 2:**
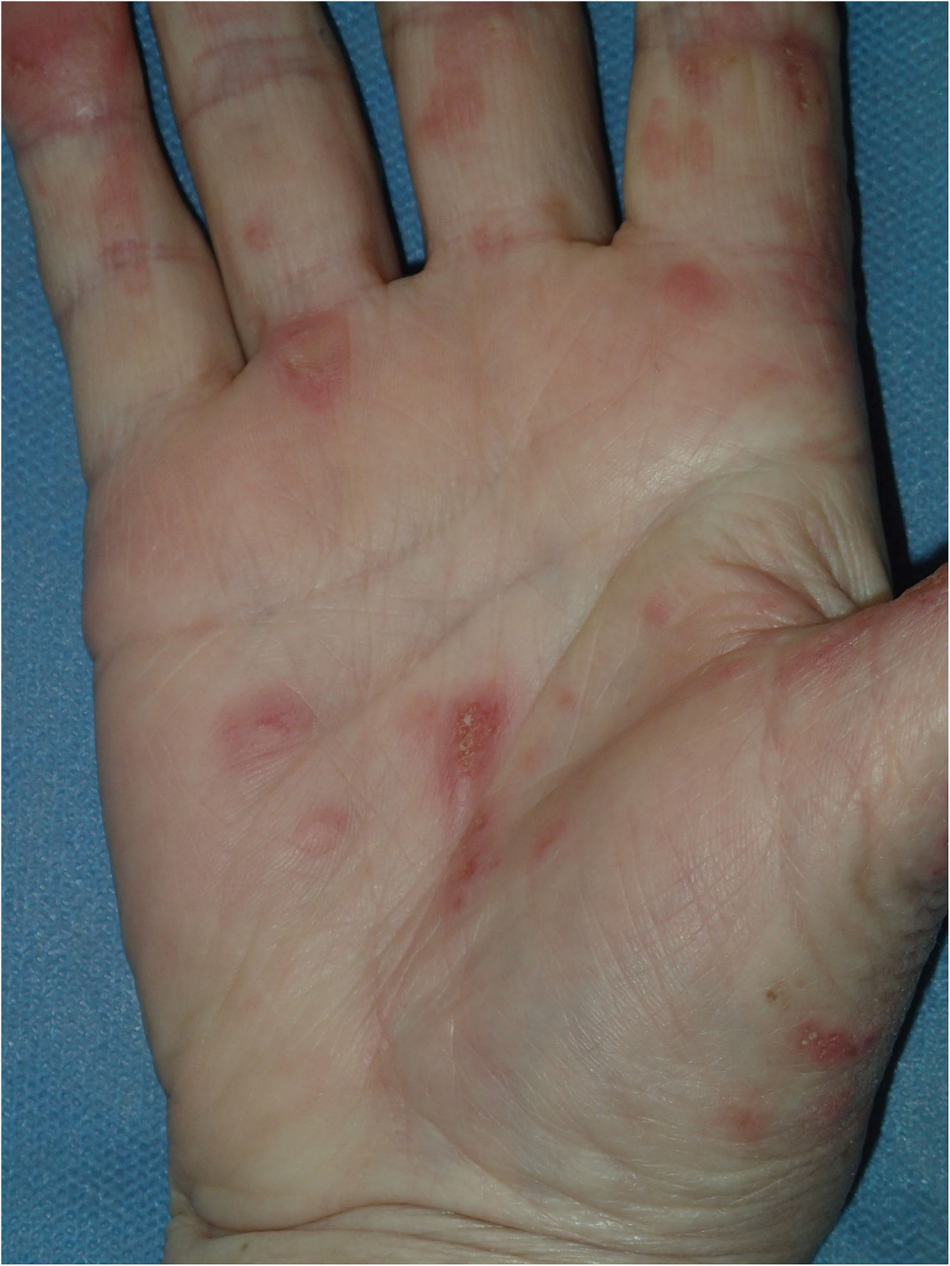
Flat targetoid erythema multiforme (EM)-like lesions on the palmar skin, compatible with Rowell syndrome.

Physical examination revealed a widespread erythematous scaly annular and polycyclic eruption symmetrically distributed on the extensor aspects of the arms, lower legs, lateral parts of the face, and scalp (Figure 1). A “trailing scale” was present at the borders of all annular plaques. In addition, multiple targetoid, erythema multiforme (EM)-like lesions were observed on the chest, back, neck, and dorsal hands, and on the lower lip vermilion (Figure 2). Diffuse hair loss and patches of cicatricial alopecia were also present upon scalp examination. Mucous membranes were not affected. Apart from the skin rash, no other systemic signs or symptoms of rheumatic disease were present.

The results of laboratory tests upon hospital admission showed a negative rapid SARS-CoV-2 antigen test and normal complete blood count, erythrocyte sedimentation rate, C-reactive protein, blood chemistry, electrolytes, liver and kidney function tests, and urinalysis. On the other hand, immune serology for lupus markers revealed positive anti-SS-A native (60 kDa) (Ro/SSA), 34 U/ml (<10 U/ml), anti-SSA (Ro-52 recombinant), 44 U/ml (<10 U/ml), and anti-ribosomal P antibodies, 63 U/ml (<10 U/ml), as well as low complement C4, 0.064 (0.20–0.65 g/l), whereas anti-La/SSB, Sm, dsDNA, RNP, anti-histone, anti-cyclic citrullinated peptide, anti-phospholipid antibodies, rheumatoid factor (RF), and immunofluorescence antinuclear antibody (ANA) test (HEp-2 substrate) were all negative. Photo testing with a standardized protocol revealed positive results for ultraviolet (UV)-A and UV-B.

**Figure 3:**
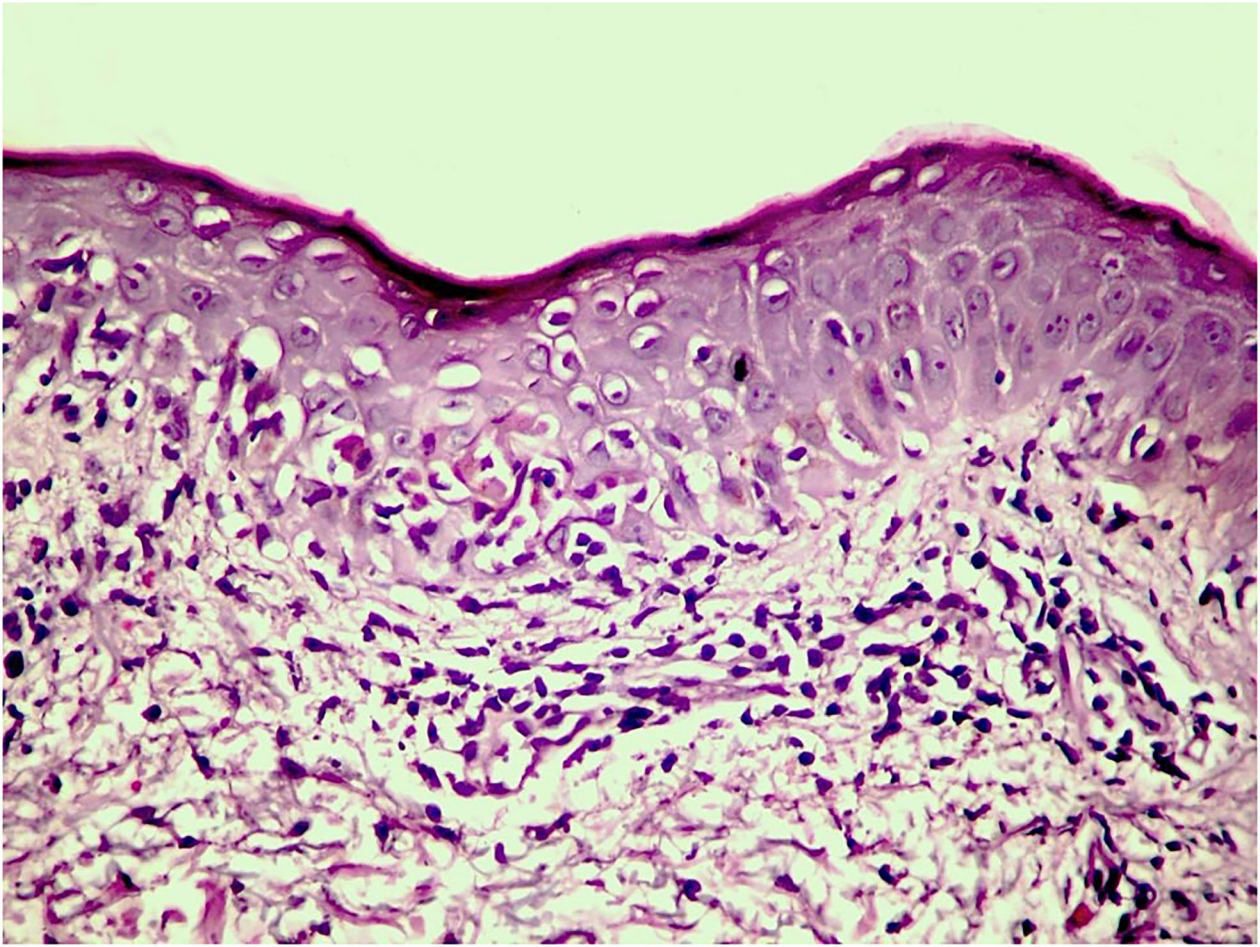
Hematoxylin and eosin stain of a biopsy from the periphery of an annular lesion showing SCLE features: epidermal atrophy, interface dermatitis, and lymphohistiocytic dermal infiltrate.

A skin biopsy from the active border of an EM-like lesion on the dorsal forearm showed epidermal atrophy and vacuolar interface dermatitis with an intense hydropic degeneration of the basal layer and few necrotic keratinocytes, as well as lymphohistiocytic infiltrate beneath the epidermis, along the dermo-epidermal junction (DEJ), together with some degree of leukocytoclasia (Figure 3).

**Figure 4:**
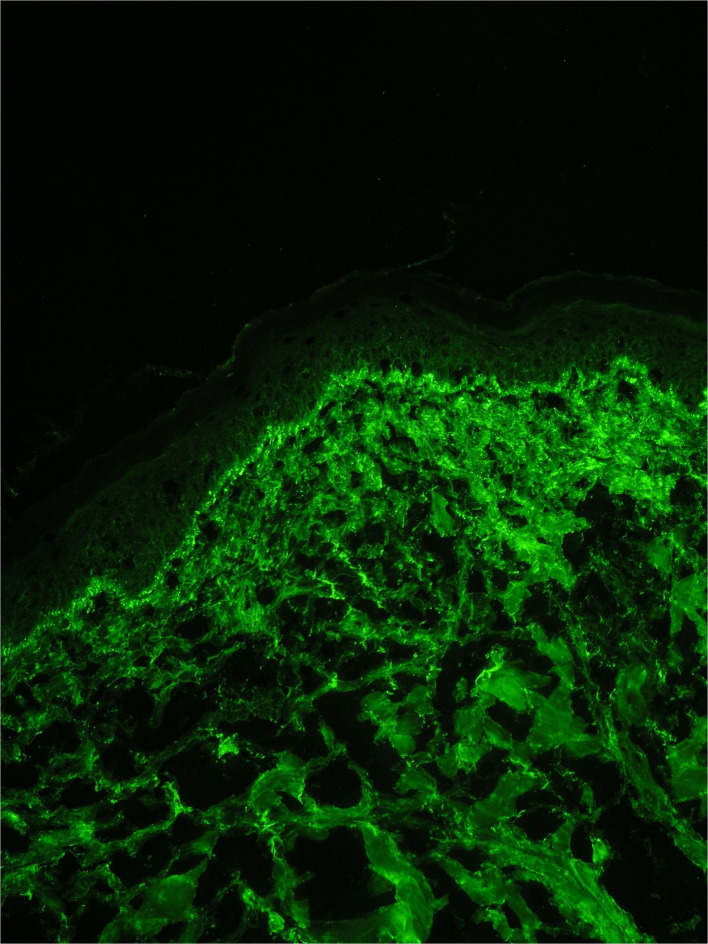
Direct immunofluorescence on a biopsy from a lesion on photo exposed skin showing a granular band of immuno-reactant deposition along the dermal-epidermal junction.

Direct immunofluorescence revealed the presence of a positive lupus band of immune reactants at the DEJ in both lesional (IgA, IgM, complement C3) and clinically uninvolved non-exposed skin (IgM) (Figure 4). Dust-like epidermal fluorescence was not found in any of the biopsy specimens.

According to the above clinical, histologic, immunologic, and serologic findings, the diagnosis of subacute cutaneous LE presenting as Rowell syndrome was established. Treatment with systemic methylprednisolone at a dose of 40 mg/daily and hydroxychloroquine 200 mg twice/daily resulted in progressive clinical improvement of the cutaneous lesions including signs of hair loss over a 2-week hospital stay. The patient was followed up for 6 months, during which period she remained in clinical remission on a maintenance corticosteroid dose of 4 mg/day, hydroxychloroquine 400 mg/day, topical corticosteroids, and photo protection.

## Discussion

The occurrence of EM-like lesions in the setting of cutaneous LE is referred to as Rowell syndrome. The latter was first described by Rowell et al. (4) as the combination of chronic discoid LE and EM-like annular lesions in the presence of typical serologic findings including positive RF, speckled pattern of ANA, and a saline extract of human tissue (anti-SJT), now known to be similar to anti-Ro/SSA antibodies. With the description of SCLE by Gilliam and Sontheimer (5) in the 1970s, it became increasingly clear that the latter can present with several unusual clinical subtypes, such as erythrodermic, acral, vitiligo-like, or poikilodermatous SCLE (6, 7) and SCLE with EM-like lesions (8). The existence of Rowell syndrome as a distinct entity has been therefore questioned and it was attributed to the diverse clinical spectrum of SCLE and considered to be rather a limited form of expression of SCLE with EM-like, or Stevens-Johnson Syndrome (SJS)/Toxic Epidermal Necrolysis (TEN)-like lesions (9). Currently, Rowell syndrome is widely considered to be a variant of SCLE (10).

Our patient met the diagnostic criteria for Rowell syndrome, i.e. occurrence of a photo-distributed annular-polycyclic eruption together with EM-like lesions, and positive anti-Ro/SSA and anti-Ro-52 antibodies present in up to 90% of SCLE cases (7). The negative RF, on the other hand, does not rule out the diagnosis since it has been found in less than half of the published cases of Rowell syndrome. The diagnostic significance of anti-Ro/SSA antibodies is much higher for SCLE because they have been found in more than two-thirds of a large cohort of patients with SCLE, while anti-La/SSB antibodies were only present in one-third of them (11). In addition, the possibility of EM merely occurring in a patient with CLE following SARS-CoV-2 infection was ruled out in our patient based on the clinical, histopathologic, and immunofluorescence findings that strongly supported the diagnosis of SCLE.

It is commonly recognized that SCLE skin lesions and Ro/SS-A autoantibody production can be triggered by a number of drugs, the majority of which are capable of producing photosensitivity drug reactions (12). The past medical history of the patient described herein, including the treatment received for COVID-19, did not include any of the drugs reported to induce SCLE (13). Other reported eliciting/exacerbating factors include cigarette smoking, psychological stress, and infection (12), the latter of which merits attention in this case.

Various pathogens have been implicated in the pathogenesis of systemic LE (SLE), namely, viruses, such as human endogenous retroviruses, parvovirus B19, herpes-zoster virus, cytomegalovirus, human immunodeficiency virus type 1, hepatitis A and C virus, rubella virus, and recently, coronaviruses (14–17).

The occurrence of several autoimmune diseases has been described secondary to COVID-19 including Guillian-Barré syndrome, immune thrombocytopenia, Miller Fischer syndrome, anti-phospholipid syndrome, type 1 diabetes mellitus, and Kawasaki disease-like syndrome (16, 18). There is also an increasing number of reports published in the literature of SLE developed after COVID-19 (19–22). Exposure to foreign peptides homologous to human peptides, i.e. molecular mimicry between the virus and human peptides, has been proposed as the main cause of the autoimmune phenomena observed in SARS-CoV-2 infection during which the immune responses raised against the virus may cross-react with human proteins that share peptide sequences with the virus leading to autoimmune pathologic sequelae. Epigenetic dysregulation of angiotensin-converting enzyme 2 and interferon (IFN)-regulated genes has been suggested to increase the sensitivity to SARS-CoV-2 in patients with lupus and to lead to new flares (3). COVID-19 infection causes a dysregulated cytokine response with a high resultant expression of IFN-gamma and pro-inflammatory cytokines, such as interleukin (IL)-1, IL-6, IL-7, IL-10, and tumor necrosis factor-alpha, which in turn could potentially be exacerbated by the shift in Th1 to Th2 response seen in SLE (16). Various autoantibodies have been reported in the serum of patients with COVID-19, including anti-nuclear antibodies, such as anti−52 kDa SSA/Ro and anti−60 kDa SSA/Ro, and various anti-phospholipid antibodies (23). It is interesting to point out that Ro52 is an IFN-inducible protein, and it is also induced by viral infection or Toll-like receptor (TLR) engagement *via* type I IFN induction (24). In contrast to SLE, there are limited reports of CLE in association with SARS-CoV-2 infection. A case of chilblain LE has been described to occur in a previously healthy 24-year-old man after COVID-19 3 months earlier (25). The worsening of SCLE with the enlargement of pre-existing plaques on the trunk and emergence of new lesions has been observed in a 50-year-old woman with positive SARS-CoV-2 PCR (26). In addition, cases of Rowell syndrome and SCLE have been reported after COVID-19 vaccines but only a few reports incriminate the infection itself as a potential exacerbating factor (27–30). Based on the clinical course of the disease, we suggest that the observed Rowell syndrome-like flare-up of CLE was related to the COVID-19 infection in our patient.

## Author Contributions

All authors listed have made a substantial, direct, and intellectual contribution to the work and approved it for publication.

## Conflict of Interest

The authors declare that the research was conducted in the absence of any commercial or financial relationships that could be construed as a potential conflict of interest.

## Publisher's Note

All claims expressed in this article are solely those of the authors and do not necessarily represent those of their affiliated organizations, or those of the publisher, the editors and the reviewers. Any product that may be evaluated in this article, or claim that may be made by its manufacturer, is not guaranteed or endorsed by the publisher.
